# Characteristics and Mutational Hotspots of Plastomes in *Debregeasia* (Urticaceae)

**DOI:** 10.3389/fgene.2020.00729

**Published:** 2020-07-08

**Authors:** Ruo-Nan Wang, Richard I. Milne, Xin-Yu Du, Jie Liu, Zeng-Yuan Wu

**Affiliations:** ^1^Germplasm Bank of Wild Species, Kunming Institute of Botany, Chinese Academy of Sciences, Kunming, China; ^2^CAS Key Laboratory for Plant Diversity and Biogeography of East Asia, Kunming Institute of Botany, Chinese Academy of Sciences, Kunming, China; ^3^Key Laboratory of Resource Biology and Biotechnology in Western China, Ministry of Education, College of Life Sciences, Northwest University, Xi’an, China; ^4^Institute of Molecular Plant Sciences, School of Biological Sciences, The University of Edinburgh, Edinburgh, United Kingdom

**Keywords:** *Debregeasia*, DNA barcode, plastome phylogenomics, phylogenetic relationship, mutational hotspots, Urticaceae

## Abstract

*Debregeasia* is an economically important genus of the nettle family (Urticaceae). Previous systematic studies based on morphology, or using up to four plastome regions, have not satisfactorily resolved relationships within the genus. Here, we report 25 new plastomes for Urticaceae, including 12 plastomes from five *Debregeasia* species and 13 plastomes from other genera. Together with the one published plastome for *Debregeasia*, we analyzed plastome structure and character, identified mutation hotspots and loci under selection, and constructed phylogenies. The plastomes of *Debregeasia* were found to be very conservative, with a size from 155,743 bp to 156,065 bp, and no structural variation. Eleven mutation hotspots were identified, including three (*rpoB*-*trnC*-*GCA*, *trnT*-*GGU*-*psbD* and *ycf1*) that are highly variable both within *Debregeasia* and among genera; these show high potential value for future DNA barcoding, population genetics and phylogenetic reconstruction. Selection pressure analysis revealed nine genes (*clpP*, *ndhF*, *petB*, *psbA*, *psbK*, *rbcL*, *rpl23*, *ycf2*, and *ycf1*) that may experience positive selection. Phylogenomic analyses results suggest that *Debregeasia* was monophyletic, and closest to *Boehmeria* among genera examined. Within *Debregeasia*, *D. longifolia* was sister to *D. saeneb*, whereas *D. elliptica*, *D. orientalis* with *D. squamata* formed the other subclade. This study enriches organelle genome resources for Urticaceae, and highlights the utility of plastome data for detecting mutation hotspots for evolutionary and systematic analysis.

## Introduction

Chloroplasts are vital organelles within plants (Raubeson and Jansen, 2005), and their genomes comprise 120 kb to 160 kb of often highly conserved DNA and gene sequences (Wicke et al., 2011), providing rich resources for the study of evolution, DNA barcoding, taxonomy and phylogeny (Borsch and Quandt, 2009; Dong et al., 2012; Ruhfel et al., 2014). Over the past decade, analysis of whole plastomes and/or protein-coding genes has been used successfully to address phylogenetic relationships at multiple taxonomic levels (e.g., Ma et al., 2014; Du et al., 2017; Li H. T. et al., 2019). Repeating sequences can cause structural changes in genomes, and because of their variability between and within lineages, they can be used to study the population genetics of taxa (Timme et al., 2007), such as in *Aristolochia* (Li X. et al., 2019); they can also serve as information regions for developing genomic markers for phylogenetic analysis, including taxonomically challenging species complexes. Such repeating markers include simple sequence repeats (SSRs), known as microsatellites, which comprise 1–6 nucleotide repeat units and are ubiquitous in the genome (Powell et al., 1996). Certain genes exhibit high variability, especially *ycf1*, which can therefore potentially be used as a barcode for terrestrial plants (Dong et al., 2015), and *rpl20*, which has an important role in protein synthesis and is involved in protein translation (Weglöhner and Subramanian, 1992). Furthermore, understanding plastome genetic variation within and between populations provides important information that can be used for conserving species and populations, helping them adapt to climate and habitat changes, and for more successful plant breeding (Daniell et al., 2016). Combining genome-wide information with that from hyper-variable regions provides the best approach to elucidate relationships and identify species among taxonomically critical groups (e.g., Bi et al., 2018; Fu et al., 2019).

*Debregeasia* Gaud. (Urticaceae) occurs mostly in East Asia, and comprises about eight species (Chen et al., 2003; Wilmot-Dear and Friis, 2012). *Debregeasia* is economically important because of its stem fibers, which are usually used to make ropes and fishing nets, and its edible fruits can be used to make wines (Chen et al., 2003). Additionally, *Debregeasia* has been used to treat diarrhea, bone fractures, tumors, skin diseases and urinary complaints, and contains compounds with anti-bacterial, immune suppressant, anti-fungal and anti-inflammatory properties (Akbar and Malik, 2002; Almubayedh and Ahmad, 2019). Thus far, morphology-based taxonomic treatments for *Debregeasia* have been controversial (Chen et al., 2003; Wilmot-Dear and Friis, 2012), whereas phylogenetic analyses have so far used too few loci to achieve full resolution within *Debregeasia* (Wu et al., 2013, 2018). Therefore, new methods based on plastome genomic data need to be explored to study the systematics of *Debregeasia*. However, only one plastome (*D. orientalis*) has been reported in *Debregeasia* (Wang et al., 2019), and neither plastome characteristics nor mutation hotspots have so far been investigated in the genus.

In the present study, a total of 25 complete plastomes of Urticaceae were newly assembled and annotated (including 12 individuals from 5 *Debregeasia* species). Together with the one published plastome, these were used to: (1) analyze variation in genome size, content and structure, as well as IR contraction and expansion; (2) identify microsatellite types, hotspot regions for sequence divergence and variation and adaptive selection; (3) reconstruct phylogenetic relationships of *Debregeasia*. The present study therefore enriches organelle genome resources for Urticaceae.

## Materials and Methods

### Plant Material

Leaf materials were collected from healthy living plants in the field, and subsequently dried and stored in silica gel. In addition, a few individuals were sampled from herbarium specimens. In total, thirteen individuals of five *Debregeasia* species were included (Supplementary Table S1), all newly sequenced except for *Debregeasia orientalis*_LAD10 (MH196364) (Wang et al., 2019) which was downloaded from GenBank. An additional 13 species within Urticaceae, which represented all four main clades of the family (Wu et al., 2013, 2018) were adopted as outgroups (Table 1). All voucher specimens were deposited in the herbarium of Kunming Institute of Botany, Chinese Academy of Sciences (KUN); Royal Botanic Garden, Edinburgh (E); and Royal Botanic Gardens, Kew (K) (Supplementary Table S1).

**TABLE 1:** Comparison of plastomes features in *Debregeasia* and other Urticaceae species examined in this study.

Species	Genome size (bp)	LSC length (bp)	SSC length (bp)	IR length (bp)	Number of genes	Number of protein-coding genes	Number of tRNAs genes	Number of rRNAs genes	GC content (%)	GC content in LSC (%)	GC content in SSC (%)	GC content in IR (%)	Accession number
*Debregeasia elliptica_*De07	155,921	85,519	19,074	25,664	129 (17)	84 (6)	37 (7)	8 (4)	36.4	34.0	29.4	42.7	MN189947
*Debregeasia elliptica_*De19	155,940	85,362	19,074	25,664	129 (17)	84 (6)	37 (7)	8 (4)	36.3	34.0	29.4	42.7	MN189948
*Debregeasia longifolia_*MBD01	155,904	85,627	18,979	25,649	129 (17)	84 (6)	37 (7)	8 (4)	36.3	34.0	29.4	42.6	MN189949
*Debregeasia longifolia_*MGD09	155,809	85,535	18,976	25,649	129 (17)	84 (6)	37 (7)	8 (4)	36.3	34.0	29.4	42.6	MN189950
*Debregeasia longifolia*_SDS11	155,853	85,586	18,969	25,649	129 (17)	84 (6)	37 (7)	8 (4)	36.3	34.0	29.4	42.6	MN189951
*Debregeasia longifolia*_XSJD10	155,810	85,550	18,962	25,649	129 (17)	84 (6)	37 (7)	8 (4)	36.3	34.0	29.4	42.6	MN189952
*Debregeasia orientalis*_GMD13	155,953	85,617	19,062	25,637	129 (17)	84 (6)	37 (7)	8 (4)	36.3	34.0	29.4	42.7	MN189953
*Debregeasia orientalis*_LAD10	155,920	85,584	19,062	25,637	129 (17)	84 (6)	37 (7)	8 (4)	36.3	34.0	29.4	42.7	MH196364
*Debregeasia orientalis*_MK05	155,939	85,545	19,066	25,664	129 (17)	84 (6)	37 (7)	8 (4)	36.3	34.0	29.4	42.7	MN189955
*Debregeasia orientalis*_ZXD12	155,992	85,561	19,103	25,664	129 (17)	84 (6)	37 (7)	8 (4)	36.3	34.0	29.4	42.7	MN189956
*Debregeasia saeneb*_PYD03	155,743	85,474	18,971	25,649	129 (17)	84 (6)	37 (7)	8 (4)	36.3	34.0	29.4	42.6	MN189957
*Debregeasia saeneb*_Q09	155,790	85,512	18,980	25,649	129 (17)	84 (6)	37 (7)	8 (4)	36.3	34.0	29.4	42.6	MN189958
*Debregeasia squamata*_Q05	156,065	85,649	19,088	25,664	129 (17)	84 (6)	37 (7)	8 (4)	36.3	34.0	29.4	42.7	MN189959
*Boehmeria nivea* var. *nipononivea*_B32	155,806	85,717	18,693	25,698	129 (17)	84 (6)	37 (7)	8 (4)	36.4	34.0	29.8	42.6	MN189944
*Boehmeria tomentosa*_B38	154,938	85,720	17,822	25,698	128 (17)	84 (6)	36 (7)	8 (4)	36.4	34.0	29.9	42.6	MN189945
*Cecropia pachystachya*_B5	153,655	84,645	18,124	25,443	129 (17)	84 (6)	37 (7)	8 (4)	36.6	34.1	30.4	42.8	MN189946
*Droguetia iners*_Dr4	149,414	81,326	17,748	25,170	128 (17)	84 (6)	36 (7)	8 (4)	36.9	35.7	30.3	42.8	MN189960
*Elatostema laevissimum* var. *laevissimum*_E36	150,244	83,968	17,118	24,579	129 (17)	84 (6)	37 (7)	8 (4)	36.2	33.7	29.5	43.0	MN189961
*Gonostegia hirta*_Go1	159,085	78,970	18,661	30,727	136 (24)	91 (13)	37 (7)	8 (4)	35.9	33.8	29.3	40.6	MN189962
*Hemistylus odontophylla*_W275	153,652	84,346	18,732	25,287	129 (17)	84 (6)	37 (7)	8 (4)	36.0	33.6	28.9	42.6	MN189963
*Hesperocnide tenella_*W277	146,844	79,535	17,692	24,808	130 (19)	84 (7)	38 (8)	8 (4)	36.4	33.9	29.7	42.7	MN189964
*Oreocnide frutescens*_GLGE12243	156,966	86,562	19,016	25,694	129 (17)	84 (6)	37 (7)	8 (4)	36.3	34.0	29.5	42.7	MN189965
*Parietaria debilis*_Pa1	152,988	84,424	18,712	24,926	129 (17)	84 (6)	37 (7)	8 (4)	36.2	34.0	29.1	42.7	MN189966
*Pipturus arborescens*_pip10	154,069	84,767	18,696	25,303	129 (17)	84 (6)	37 (7)	8 (4)	36.2	33.9	29.3	42.7	MN189967
*Pouzolzia sanguinea* var. *elegans*_Po11	153,715	84,158	18,701	25,428	129 (17)	84 (6)	37 (7)	8 (4)	36.3	34.1	29.3	42.7	MN189968
*Rousselia humilis*_W142	153,301	84,334	18,505	25,231	129 (17)	84 (6)	37 (7)	8 (4)	36.0	33.6	29.0	42.6	MN189969

*The numbers in parenthesis indicate the genes duplicated in the IR regions.*

### DNA Extraction, Sequencing, Plastomes Assembly and Annotation

For silica gel dried materials, DNA was extracted using a modified hexadecyltrimethylammonium bromide (CTAB) method (Doyle and Doyle, 1987), whereas for herbarium specimens, DNA was extracted using Tiangen DNA secure Plant Kits (DP320) (Tiangen Biotech, Beijing, China). The quality and quantity of DNA were measured on 1% Tris–acetate–ethylenediamine tetraacetic acid (TAE) agarose gels and using fluorometric quantification on the Qubit (Invitrogen, Carlsbad, California, United States). Paired-end libraries with 500 bp insert-size were prepared and then sequenced using the Illumina HiSeq X Ten platform, the length of reads was 150 bp. A total of 2 to 4 Gb clean data were generated for each individual. *De novo* assemblies were constructed with Spades (Bankevich et al., 2012). GetOrganelle v1.7.0 (Jin et al., 2018) was used to improve accuracy and efficiency in *de novo* assembly. Reference-guided connecting was subsequently conducted using Bandage (Wick et al., 2015) and Geneious v8.1 (Kearse et al., 2012), to generate circular plastomes. The newly generated genomes were automatically annotated by PGA (Qu et al., 2019), then adjusted and confirmed using Geneious. The patterns of genomic variation among the plastomes were calculated and visualized using OGDRAW v1.3.1 (Greiner et al., 2019) and Circos v0.69-9 (Krzywinski et al., 2009).

### Repeat Sequence Analysis

REPuter (Kurtz et al., 2001) was used to identify dispersed (including forward, reverse and complement repeat sequences) and palindrome repeat sequences according to the following settings: sequence identity was 90%, Hamming distance equal to 3, the minimum repeat size was 30 bp and the maximum computed repeats was 100. The tandem repeats were identified using the online Tandem Repeats Finder (Benson, 1999). The alignment parameters match, mismatch, and indels were 2, 7, and 7, respectively. The minimum alignment score to report repeats was 80. The maximum period size and TR array size were limited to 500 bp and two million bp, respectively. ESTs (Thiel et al., 2003) was used to identify simple sequence repeats (SSRs) with the minimum repeat number set to 10, 5, 4, 3, 3, and 3 for mono-, di-, tri-, tetra-, penta- and hexa-nucleotides, respectively.

### Estimation of Sequence Divergence and Mutational Hotspots

In order to determine the structure and sequence divergence of the plastomes of *Debregeasia*, we used the Mauve alignment tool embedded in Geneious, and the VISTA framework (Frazer et al., 2004) to compare the 13 plastomes. The boundaries between the IR and SC regions of these were compared and analyzed. Individual coding and non-coding regions were extracted by Geneious, and homologous loci were aligned using MAFFT v1.3.3 (Katoh et al., 2002). Then we determined the percentage of variable sites, calculated thus: (number of nucleotide substitutions + number of indels) / (length of aligned sites minus length of indels + number of indels) * 100%. Following this, the seven regions with the highest mutation rate were identified as mutation hotspots for *Debregeasia*. Due to the over-conserved genomic structure of *Debregeasia* plastomes, we compared in a similar way the 13 outgroup species, with each other and with *Debregeasia*, to investigate plastome structures and sequence divergence across Urticaceae, and hence identified the seven most variable regions at family level.

### Positive Selection Tests

Non-synonymous (dN) and synonymous (dS) nucleotide substitution rates, as well as their ratios (w = dN/dS) were analyzed using Codeml (PAML v4.7) (Yang and Nielsen, 2002; Yang, 2007). The protein-coding genes were extracted and aligned using MAFFT. Six site-specific models (M0, M1, M2, M3, M7, and M8) were applied, to identify the selection pressure across plastomes. These models allowed the ω ratio to vary among sites, with a fixed ω ratio in all the branches. The dN, dS, and ω values were calculated with Codeml (seqtype = 1, model = 0, NSsites = 0, 1, 2, 3, 7, 8). Then we compared pairs of site-specific models as follows: M0 (one-ratio) vs. M3 (discrete), M1 (nearly neutral) vs. M2 (positive selection) and M7 (β) vs. M8 (β and ω) to analyze the existence of positive selection, with *p* values for each comparison determined via a Likelihood ratio test (LRT). Bayes Empirical Bayes inferences were calculated in site models M2 and M8 to estimate the posterior probabilities and positive selection pressures of the selected genes.

### Phylogenetic Analysis

Phylogenetic relationships of the examined *Debregeasia* species, plus 13 outgroup species, were analyzed using four datasets, all based on plastome data. These were (a) complete plastomes, (b) plastome protein-coding genes, (c) those mutational hotspots identified that were among the seven most variable at both genus and family level (i.e., *rpoB*-*trnC*-*GCA*, *trnT*-*GGU*-*psbD*, and *ycf1*), and (d) those mutational hotspots identified that were among the seven most variable at genus level, or at family level, or both (i.e., *psbK*-*psbI*, *rpl36*-*rps8*, *rpoB*-*trnC*-*GCA*, *trnK*-*UUU*-*rps16*-*trnQ*-*UUG*, *trnP*-*UGG*-*psaJ*, *trnT*-*GGU*-*psbD*, *trnT*-*UGU*-*trnL*-*UAA*, *ycf4*-*cemA*, *matK*, *ndhF*, and *ycf1*). The datasets were aligned with MAFFT. The best substitution model (TVM+G) was determined by the Bayesian information criterion (BIC) in jModelTest2 (Darriba et al., 2012). Maximum likelihood (ML) analyses were performed using RAxML v2.0.1 (Stamatakis, 2006) with 1000 bootstrap replicates. Maximum Parsimony (MP) phylogenetic trees were constructed using MEGA v7.0 (Kumar et al., 2016). Bayesian inference (BI) was carried out by MrBayes v3.2 (Ronquist et al., 2012) at the CIPRES Science Gateway v3.3 (Miller et al., 2010). One-million-generation iterations were performed, with trees being sampled every 200 generations, with four runs, each with four chains run in parallel. The Markov Chain Monte Carlo (MCMC) output (infile.nex.run1.p files) was examined to check convergence and to ensure that all the Effective Sample Sizes (ESS) values were above 200. Figtree v1.4 (Rambaut, 2012) was used to visualize and annotate the output trees.

## Results

### Plastome Structures

**FIGURE 1:**
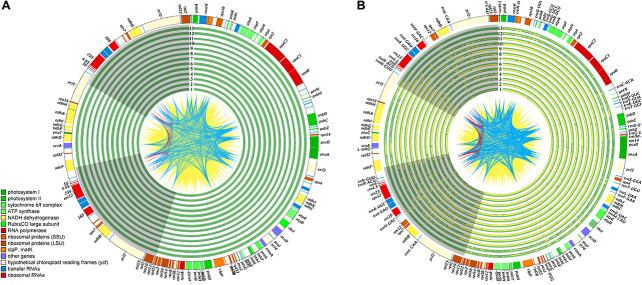
An overview of plastome variation. In the quadripartite structure of these plastomes, the two IR regions (IRa and IRb) are shown with gray background, while the large and small single-copy regions (LSC and SSC) are displayed with blank background. The lines, CDS to CDS, are filled with yellow ridges while the tRNA to tRNA are occupied with blue ridges, and other red lines are rRNA to rRNA. The identical sites is filled with green ridges while the variations are occupied with yellow ridges. **(A)** An overview of plastome variation across the *Debregeasia*, with *D. elliptica*_De07 as reference. The studied *Debregeasia* species are indicated as in Supplementary Table S6. **(B)** An overview of plastome variation across the Urticaceae, with *D. elliptica*_De07 as reference. The sampled Urticaceae species information are listed in Supplementary Table S7.

The plastomes of all five *Debregeasia* species had a typical quadripartite structure, comprising a large single-copy (LSC) region and a small single-copy (SSC) region separated by a pair of inverted repeats (IRa and IRb) (Figure 1). The total length of the plastomes of these five species ranged from 155,743 bp (*D. saeneb*_PYD03) to 156,065 bp (*D. squamata*_Q05). The length of the LSC region ranged from 85,362 bp (*D. elliptica*_De19) to 85,649 (*D. squamata*_Q05), whereas that of the SSC region ranged from 18,962 bp (*D. longifolia*_XSJD10) to 19,103 bp (*D. orientalis*_ZXD12). The two IR regions had identical lengths within any individual, ranging from 25,637 bp (*D. orientalis*_GMD13 and *D. orientalis*_LAD10) to 25,664 bp (*D. elliptica*_De07, *D. elliptica*_De19, *D. orientalis*_MK05, *D. orientalis*_ZXD12, and *D. squamata*_Q05). For full details, plus those for the 13 outgroup species, see Table 1.

A total of 129 genes were identified, comprising 84 protein coding genes, 37 tRNA genes and 8 rRNA genes. Of these, 17 genes (6 protein coding genes, 7 tRNA genes and 4 rRNA genes) were duplicated in the IR regions in all *Debregeasia* species (Tables 1, 2). The gene *rps19* crossed both the LSC and IRb regions (Supplementary Figure S1), whereas both *ndhF* and *ycf1* were situated in the SSC but crossed the two IR regions in different directions. Eighteen genes had introns, among which fifteen genes (*atpF*, *ndhA*, *ndhB*, *petB*, *petD*, *rpl2*, *rpl16*, *rpoC1*, *rps16*, *trnA*-*UGC*, *trnG*-*UCC*, *trnI*-*GAU*, *trnK*-*UUU*, *trnL*-*UAA*, and *trnV*-*UAC*) contained a single intron, whereas three (*clpP*, *rps12*, and *ycf3*) contained two introns (Table 2).

**TABLE 2:** List of genes present in the plastomes of five *Debregeasia* species.

Category of genes	Group of gene	Name of gene				
Self-replication	Ribosomal RNA genes	*rrn16*^(×2)^	*rrn23*^(×2)^	*rrn4.5*^(×2)^	*rrn5*^(×2)^	
	Transfer RNA genes	trnA-UGC*^(×2)^	*trnC-GCA*	*trnD*-GUC	*trnE-UUC*	*trnF-GAA*
		*trnfM*-*CAU*	*trnG-GCC*	trnG-UCC*	*trnH-GUG*	*trnI-CAU*^(×2)^
		trnI-GAU*^(×2)^	trnK-UUU*	*trnL-CAA*^(×2)^	trnL-UAA*	*trnL-UAG*
		*trnM-CAU*	*trnN-GUU*^(×2)^	*trnP-UGG*	*trnQ-UUG*	*trnR-ACG*^(×2)^
		*trnR-UCU*	*trnS-GCU*	*trnS-GGA*	*trnS-UGA*	*trnT-GGU*
		*trnT-UGU*	*trnV-GAC*^(×2)^	trnV-UAC*	*trnW-CCA*	*trnY-GUA*
	Small subunit of ribosome	*rps2*	*rps3*	*rps4*	*rps7*^(×2)^	*rps8*
		*rps11*	rps12**^(×2)^	*rps14*	*rps15*	rps16*
		*rps18*	*rps19*			
	Large subunit of ribosome	rpl2*^(×2)^	*rpl14*	rpl16*	*rpl20*	*rpl22*
		*rpl23*^(×2)^	*rpl32*	*rpl33*	*rpl36*	
	DNA-dependent RNA polymerase	*rpoA*	*rpoB*	rpoC1*	*rpoC2*	
Genes for photosynthesis	Subunits of NADH-dehydrogenase	ndhA*	ndhB*^(×2)^	*ndhC*^*a*^	*ndhD*	*ndhE*
		*ndhF*	*ndhG*	*ndhH*	*ndhI*^*acd*^	*ndhJ*
		*ndhK*^*a*^				
	Subunits of photosystem I	*psaA*	*psaB*	*psaC*	*psaI*	*psaJ*
	Subunits of photosystem II	*psbA*	*psbB*	*psbC*	*psbD*	*psbE*
		*psbF*	*psbH*	*psbI*	*psbJ*	*psbK*
		*psbL*	*psbM*	*psbN*	*psbT*	*psbZ*
	Subunits of cytochrome b/f complex	*petA*	petB*	petD*	*petG*	*petL*
		*petN*				
	Subunits of ATP synthase	*atpA*	*atpB*	*atpE*	atpF*	*atpH*
		*atpI*				
	Subunits of rubisco	*rbcL*				
Other genes	Maturase	*matK*				
	Protease	clpP**				
	Envelope membrane protein	*cemA*				
	Subunit of Acetyl-Co A-carboxylase	*accD*				
	C-type cytochrome synthesis gene	*ccsA*				
Genes of unknown function	Conserved open reading frames	*ycf1*	*ycf2*^(× 2)^	ycf3**	*ycf4*	

*Gene contains one intron; **gene contains two introns; (×2) indicates the number of the repeat unit is 2.

**FIGURE 2:**
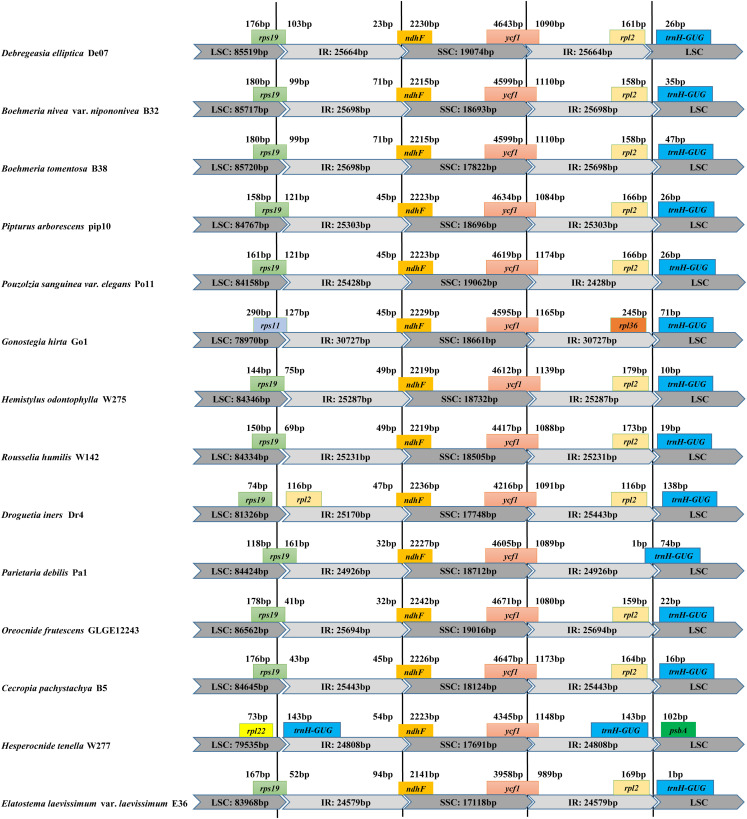
Comparison of the borders of LSC, SSC, and IR regions between the complete plastomes of *D. elliptica*_De07 and 13 other Urticaceae species.

Within *Debregeasia*, no IR contraction was observed in any plastomes, whereas IR expansion generally seemed very conservative. In outgroups, the LSC/IR and IR/SSC boundaries showed some differences from *Debregeasia* (Figure 2). In *Gonostegia hirta*_Go1, the gene *rps11* crossed from LSC to IRb, and the *rpl36* gene was near the IRa/LSC boundary. In *Droguetia iners*_Dr4, the gene *rps19* was only in the large single-copy. In *Parietaria debilis*_Pa1, the genes *rps19* and *trnH*-*GUG* crossed from the LSC to the IRb and IRa regions, respectively. In *Hesperocnide tenella*_W277, *trnH*-*GUG* was copied in both IR regions.

### Repeat Structure and Simple Sequence Repeats

**FIGURE 3:**
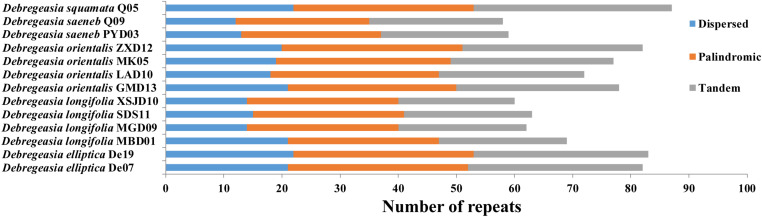
The numbers of three types of repeats in 13 individuals of five *Debregeasia* species.

A total of 932 repeats were identified in *Debregeasia*, falling into three categories (Table 3). Of these, the most frequent were palindromic repeats, which occurred 363 times (38.95%), followed by tandem repeats (337 instances, 36.16%), and dispersed repeats (forward, reverse, or complement), of which there were 232 (24.89%). The individual accession with the greatest number of repeats was *D. squamata*_Q05 with 87, comprising 22 dispersed repeats, 31 palindromic repeats, and 34 tandem repeats. The greatest numbers of dispersed, palindromic and tandem repeats were found in *D. elliptica_De19* (22), *D. elliptica*_De07 (31) and *D. squamata*_Q05 (34), respectively (Figure 3).

**TABLE 3:** The distribution of repeats across the plastomes of *Debregeasia*.

Species	Dispersed				Palindromic	Tandem	Total
	F	R	C	total			
*Debregeasia elliptica*_De07	20	1	0	21	31	30	82
*Debregeasia elliptica*_De19	21	1	0	22	31	30	83
*Debregeasia longifolia*_MBD01	19	2	0	21	26	22	69
*Debregeasia longifolia*_MGD09	12	2	0	14	26	22	62
*Debregeasia longifolia*_SDS11	14	1	0	15	26	22	63
*Debregeasia longifolia*_XSJD10	12	2	0	14	26	20	60
*Debregeasia orientalis*_GMD13	18	3	0	21	29	28	78
*Debregeasia orientalis*_LAD10	17	1	0	18	29	25	72
*Debregeasia orientalis*_MK05	18	1	0	19	30	28	77
*Debregeasia orientalis*_ZXD12	19	1	0	20	31	31	82
*Debregeasia saeneb*_PYD03	11	2	0	13	24	22	59
*Debregeasia saeneb*_Q09	11	1	0	12	23	23	58
*Debregeasia squamata*_Q05	21	1	0	22	31	34	87
Total species	213	19	0	232	363	337	932

*F: forward, R: reverse, C: complement.*

**FIGURE 4:**
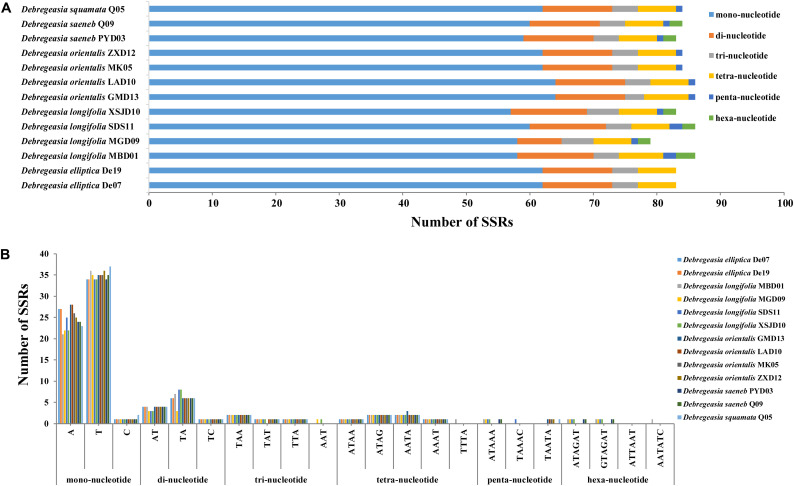
The maps of simple sequence repeats in *Debregeasia*, based on 13 individuals of five species. **(A)** The number of SSRs of each of six repeat types, categorized by number of nucleotides. **(B)** The number of SSRs by specific repeat type.

Six kinds of SSRs (mono-, di-, tri-, tetra-, penta- and hexa-nucleotide) were identified in the plastomes of *Debregeasia*, with 1,091 SSRs detected in total (Supplementary Table S2 and Figure 4). The most frequent SSRs were mononucleotides, making up 72.41% of the total, of which T, A, C and G mononucleotides comprised 41.61%, 29.51%, 1.28%, and none, respectively (Supplementary Table S3 and Figure 4). The frequency of SSRs was inversely proportional to their length, except that tetranucleotide SSRs were more common than trinucleotide SSRs. Within *D. longifolia*, the total number of SSRs varied from 79 (*D. longifolia*_MGD09) to 86 (*D. longifolia*_MBD01 and *D. longifolia*_SDS11), with *D. longifolia*_XSJD10 intermediate with 83. Within other *Debregeasia* species, number of SSRs varied by no more than two between accessions examined, so the variation in SSR number in *D. longifolia* is unusual in the genus (Figure 4).

### Sequence Divergence and Mutational Hotspots

**FIGURE 5:**
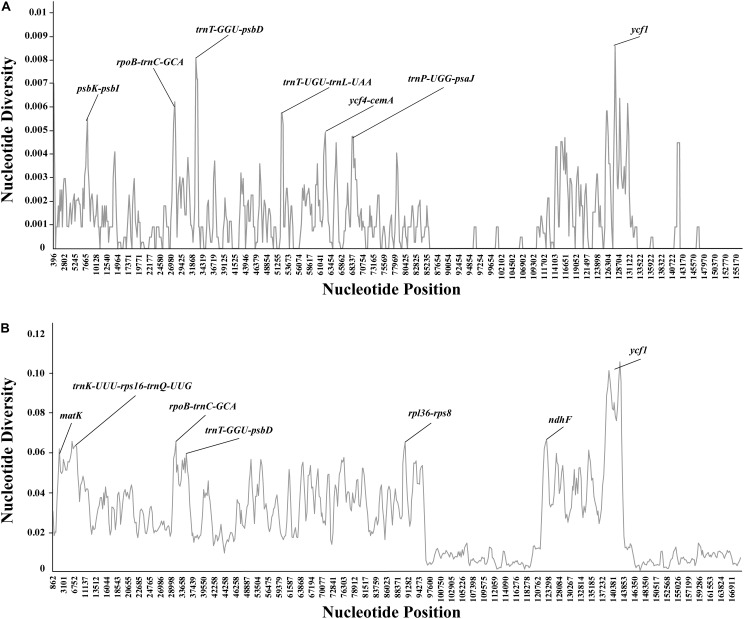
Percentages of variable sites in homologous regions of complete plastomes. **(A)** Based on 13 individuals of five *Debregeasia* species. **(B)** Based on 26 individuals of nineteen species of Urticaceae.

In general, our results showed that the plastome of *Debregeasia* is comparatively conserved, and that all genes were always present in the same order (Supplementary Figures S2, S3); this also applies across all 13 outgroup taxa (Supplementary Figure S4). Moreover, the non-coding regions had more variation, and higher levels of divergence, than the coding regions. The seven regions with the highest levels of variation were *psbK-psbI*, *rpoB-trnC*-*GCA*, *trnT-GGU-psbD*, *trnT-UGU-trnL-UAA*, *ycf4-cemA*, *trnP*-*UGG*-*psaJ*, and *ycf1*. Of these regions, *ycf1* straddled the SSC/IR boundary, whereas all of the others were located in the LSC region (Figure 5A). All had >0.5% variation across *Debregeasia* species examined. These seven regions could be considered as mutational hotspots and utilized as potential DNA barcodes for future population genetic analysis, phylogeny reconstruction and species identification studies in *Debregeasia*.

Comparing *Debregeasia* with 13 outgroup taxa, further plastome variation is notable, mainly in non-coding regions but also in the genes of *ndhF*, *ycf1* and *ycf2* (Supplementary Figure S5). The plastome sequence of *Debregeasia* is close to that of *Boehmeria*, but quite distinct from other outgroups (Supplementary Figure S5). The seven regions with highest levels of variation among genera were identified, each having >6% variation across Urticaceae genera examined. Of these regions, three (*rpoB*-*trnC*-*GCA*, *trnT*-*GGU*-*psbD*, and *ycf1*) were also among the seven most variable within *Debregeasia*, whereas four (*matK*, *trnK*-*UUU*-*rps16*-*trnQ*-*UUG*, *rpl36*-*rps8*, *ndhF*) were not (Figure 5B). Hence a total of eleven mutation hotspots, (i.e., *psbK*-*psbI*, *rpl36*-*rps8*, *rpoB*-*trnC*-*GCA*, *trnK*-*UUU*-*rps16*-*trnQ*-*UUG*, *trnP*-*UGG*-*psaJ*, *trnT*-*GGU*-*psbD*, *trnT*-*UGU*-*trnL*-*UAA*, *ycf4*-*cemA*, *matK*, *ndhF*, and *ycf1*), were identified that were highly variable within *Debregeasia* and/or across Urticaceae genera.

### Positive Selection Sites

We investigated the rate of non-synonymous (dN) and synonymous (dS) substitutions to evaluate the selective pressure for 72 common protein-coding genes among the 13 *Debregeasia* individuals examined (Supplementary Tables S4, S5), using codon substitution models to identify possible sites under positive selection. Eighteen genes with positive selection sites were identified, and these were as follows: one subunit of the Acetyl-Co A-carboxylase gene (*accD*), one C-type cytochrome synthesis gene (*ccsA*), one gene for envelope membrane protein (*cemA*), one subunit of the rubisco gene (*rbcL*), one gene for a component of the trans locus of an envelope protein (*ycf1*), one gene for photosystem I subunit (*psaB*), two subunits of ATP synthase genes (*atpA* and *atpB*), two genes for subunits of NADH-dehydrogenase (*ndhD* and *ndhF*), four genes for the synthesis of small and large ribosomal subunit proteins (*rps3*, *rps4*, *rps15*, and *rpl20*), and four DNA-dependent RNA polymerase genes (*rpoA*, *rpoB*, *rpoC1*, and *rpoC2*).

### Phylogenetic Relationships

**FIGURE 6:**
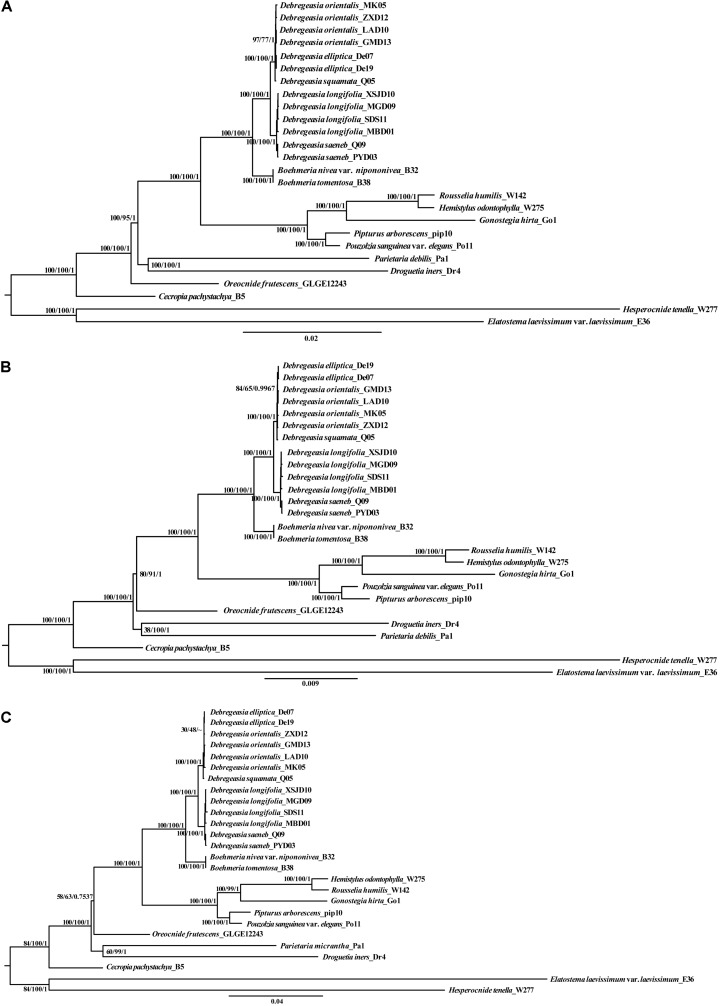
The phylogenetic relationships of five *Debregeasia* species (based on 13 individuals) plus 13 Urticaceae outgroup species, with support values shown from Maximum likelihood (ML), Maximum parsimony (MP), and Bayesian Inference (BI). Phylogenetic trees based on three datasets. **(A)** Whole plastomes; **(B)** Plastome protein-coding genes; **(C)** Three mutation hotspots at genus and family level.

Phylogenetic analysis based on five *Debregeasia* species plus 13 outgroup species, using Maximum likelihood, Maximum parsimony, and Bayesian Inference, showed that all *Debregeasia* species examined formed a single clade with high bootstrap and posterior probability support (Figure 6 and Supplementary Figure S6). The genus comprised two well-supported subclades, including *D. longifolia* plus *D. saeneb*, and *D. elliptica* plus *D. orientalis* plus *D. squamata*. The four species with multiple accessions examined were each monophyletic. Additionally, species from *Boehmeria* were resolved as the sister group to *Debregeasia*.

## Discussion

### Plastome Character and Potential Microsatellite Markers

Among the five *Debregeasia* species examined here, the plastomes appeared highly conserved, with no changes to gene order or overall structure (e.g. gene duplication, deletion and reverse transcription) observed in the genomes of *Debregeasia*. This might be because the species diverged fairly recently (Wu et al., 2015), or possibly due to the relatively conservative ecological niches of the genus.

The GC content of the LSC and SSC regions in all the *Debregeasia* species were much lower than those of the IR regions. A possible explanation for this is that the IR contains four rRNA genes, and the 16S rRNA has a very high GC content in Archaea (65–66.5%) (Yamane et al., 2011), with similar results in other terrestrial plants (Zeb et al., 2020).

Repeating sequences in plastomes can cause structural changes, and their variability across lineages makes these an appropriate source of for developing genomic markers for population genetics (Powell et al., 1996), especially when they are abundant and polymorphic. This clearly applies in *Debregeasia* and Urticaceae, wherein varying abundances of dispersed, palindromic and tandem repeats among the plastomes, both within and between species (Supplementary Table S2) may provide additional phylogenetic signals and evolutionary information. Additionally, large numbers of SSRs (Microsatellites) were detected in all plastomes of *Debregeasia*, with mononucleotide SSRs the most frequent, providing ample markers for further population and phylogenetic analysis. The number of SSRs was considerably more variable within *D. longifolia* than in *D. orientalis*, although four individuals of each were examined (Figures 3, 4 and Supplementary Table S2). Our data does not show an obvious reason for this, as *D. orientalis* shows more variation in both latitude and altitude than *D. longifolia* (Supplementary Table S2), but *D. longifolia* might exhibit greater variation in habitats occupied.

### Utility of Plastomes in Phylogenomics and DNA Barcoding

Complete plastome sequences are increasingly being used to solve taxonomic problems among closely related groups, providing valuable information for phylogenetic reconstruction (e.g., Ma et al., 2014; Dong et al., 2018; Li H. T. et al., 2019). In *Debregeasia*, phylogenetic relationships within have so far remained insufficiently resolved, probably because previous studies (Wu et al., 2015, 2018) have employed a limited number of DNA loci, providing insufficient information for full resolution. Here, the monophyly of *Debregeasia* received maximum bootstrap and Bayesian support, improving on previous studies using less data (Wu et al., 2013, 2018). Support for groupings within the genus also increased, and tree topology generally did not vary across methods or datasets, except for a few less well-supported groups at the tree tips, for example: *D. elliptica* appears nested within *D. orientalis* for some analyses and data sets, but not others (Figure 6), however, these relationships are not strongly supported. This may reflect recent divergence of these species, and hence it is possible that more intensive sampling of populations within both species, together with nuclear genomic data will provide a clearer picture in the future.

DNA super barcodes (whole genome) and mini barcodes (a proportion of a barcode) are extensions to the practice of routine DNA barcoding (Little, 2014; Hollingsworth et al., 2016). Theoretically, whole plastomes or nuclear genomes will provide the final solution for species identification. However, from both an economic and a practical perspective, a barcode or mini barcode is often sufficient, e.g., for *Taxus* (Liu et al., 2018) and macrophyte (Ortega et al., 2020) identification. In our study, the whole plastome can clearly distinguish all five *Debregeasia* species examined (Figure 6A). Meanwhile, three regions (*rpoB-trnC-GCA*, *trnT-GGU-psbD* and *ycf1*) showed high levels of variation at both within *Debregeasia* and between genus (Urticaceae) levels (percentage of variability >0.5% and >6.0%, respectively), and can distinguish all five *Debregeasia* species (Figure 6C). Indeed *ycf1*, recently proposed as the most promising plastid DNA barcode across all land plants (Dong et al., 2015), could separate all five *Debregeasia* species on its own (data not shown). These mutational hotspots have the potential to resolve taxonomic issues in the family, and for future use as barcodes and for species identification. Therefore, plastome data shows great potential for the study of evolution, taxonomy and phylogenetic relationships in the genus *Debregeasia* and elsewhere in the Urticaceae.

### Positive Selection Regions

Variation in both synonymous and non-synonymous nucleotide sites is also very useful in evolutionary studies (Ogawa et al., 1999). In this study, eighteen genes with sites under positive selection were identified (Supplementary Tables S4, S5), which is comparable to the sixteen detected in Orchidaceae (Dong et al., 2018), rather fewer than the 51 detected across 97 *Pinus* species (Zeb et al., 2020), but more than the seven detected among 22 Lythraceae species (Gu et al., 2019). Notably, the gene *ycf1* was both under positive selection, and a mutational hotspot, in *Debregeasia*. This gene is one of the largest genes in the plastome, encoding a component of the trans locus of the envelope protein *in vivo* (Drescher et al., 2000). The *ycf1* gene has been useful for phylogenetic analysis in other groups, and contains a site that is under positive selection in other plant lineages (e.g., Greiner et al., 2008; Hu et al., 2015). Our results could indicate a role for *ycf1* in speciation and habitat adaptation within *Debregeasia*. The roles of all genes under selection in the genus merit further investigation, with regard to the range of habitats occupied by *Debregeasia*, which include moist places by streams, thickets, forests in mountain valleys, and slopes of limestone mountains (Chen et al., 2003).

## Data Availability Statement

All datasets generated for this study are included in the Table 1.

## Author Contributions

JL and Z-YW conceived the work, and carried out the field work. R-NW, Z-YW, X-YD, and JL analyzed the data. R-NW drafted the manuscript. RM, JL, and Z-YW revised the manuscript. All authors approved the final manuscript.

## Conflict of Interest

The authors declare that the research was conducted in the absence of any commercial or financial relationships that could be construed as a potential conflict of interest.

## Supplementary Material

FIGURE S1 Comparison of the borders of LSC, SSC, and IR regions in *Debregeasia*, based on 13 individuals of five species.

FIGURE S2 Sequence identity plot comparing the plastomes based on 13 individuals of five *Debregeasia* species using mVISTA with *D. elliptica*_De07 as a reference. Genome regions are color coded as protein coding, rRNA coding, tRNA coding or conserved non-coding sequences.

FIGURE S3 MAUVE alignment of plastomes, based on 13 individuals of five *Debregeasia* species with *D. elliptica*_De07 as a reference.

FIGURE S4 MAUVE alignment of plastomes, based on 13 Urticaceae outgroup species, aligned with *D. elliptica*_De07 as a reference.

FIGURE S5 Sequence identity plot comparing the plastomes of 13 Urticaceae outgroup species using mVISTA with *D. elliptica*_De07 as a reference. Genome regions are color coded as protein coding, rRNA coding, tRNA coding or conserved non-coding sequences.

FIGURE S6 Phylogenetic relationships based on eleven mutational hotspots in five *Debregeasia* species (based on 13 individuals) and 13 Urticaceae outgroup species, with support values shown from Maximum likelihood (ML), Maximum parsimony (MP), to Bayesian Inference (BI).

TABLE S1 Sampled species and their voucher specimens used in this study.

TABLE S2 The number of simple sequence repeats (SSRs) in each *Debregeasia* plastome examined.

TABLE S3 The subtypes of each of the six SSRs categories detected in *Debregeasia* plastomes.

TABLE S4 Maximum likelihood parameter estimates for 78 genes of the *Debregeasia* species examined.

TABLE S5 Likelihood ratio test (LRT) of the variable ω ratio under different models.

TABLE S6 The studied *Debregeasia* species are indicated in Figure 1A.

TABLE S7 The studied Urticaceae species are indicated in Figure 1B.
