# Supplementary figures and images for: Characteristics and Mutational Hotspots of Plastomes in Debregeasia (Urticaceae)
Source: Front Genet. 2020 Jul 8;11:729. doi: 10.3389/fgene.2020.00729 (PMC7360830; doi:10.3389/fgene.2020.00729)

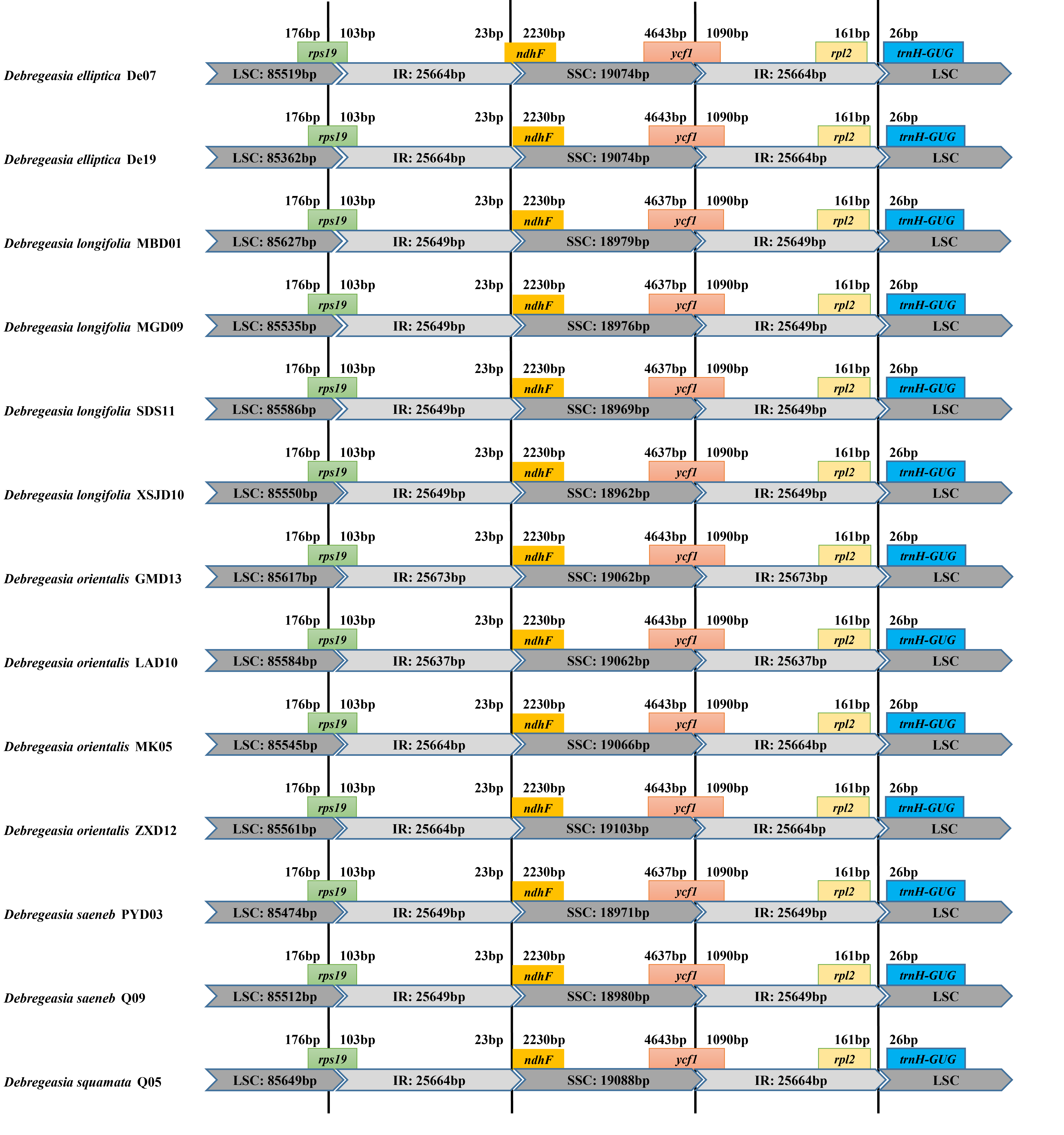

Supplement: FIGURE S1 — Comparison of the borders of LSC, SSC, and IR regions in Debregeasia, based on 13 individuals of five species. [file Data_Sheet_1.zip › supplementary files/Figure S1.tif]

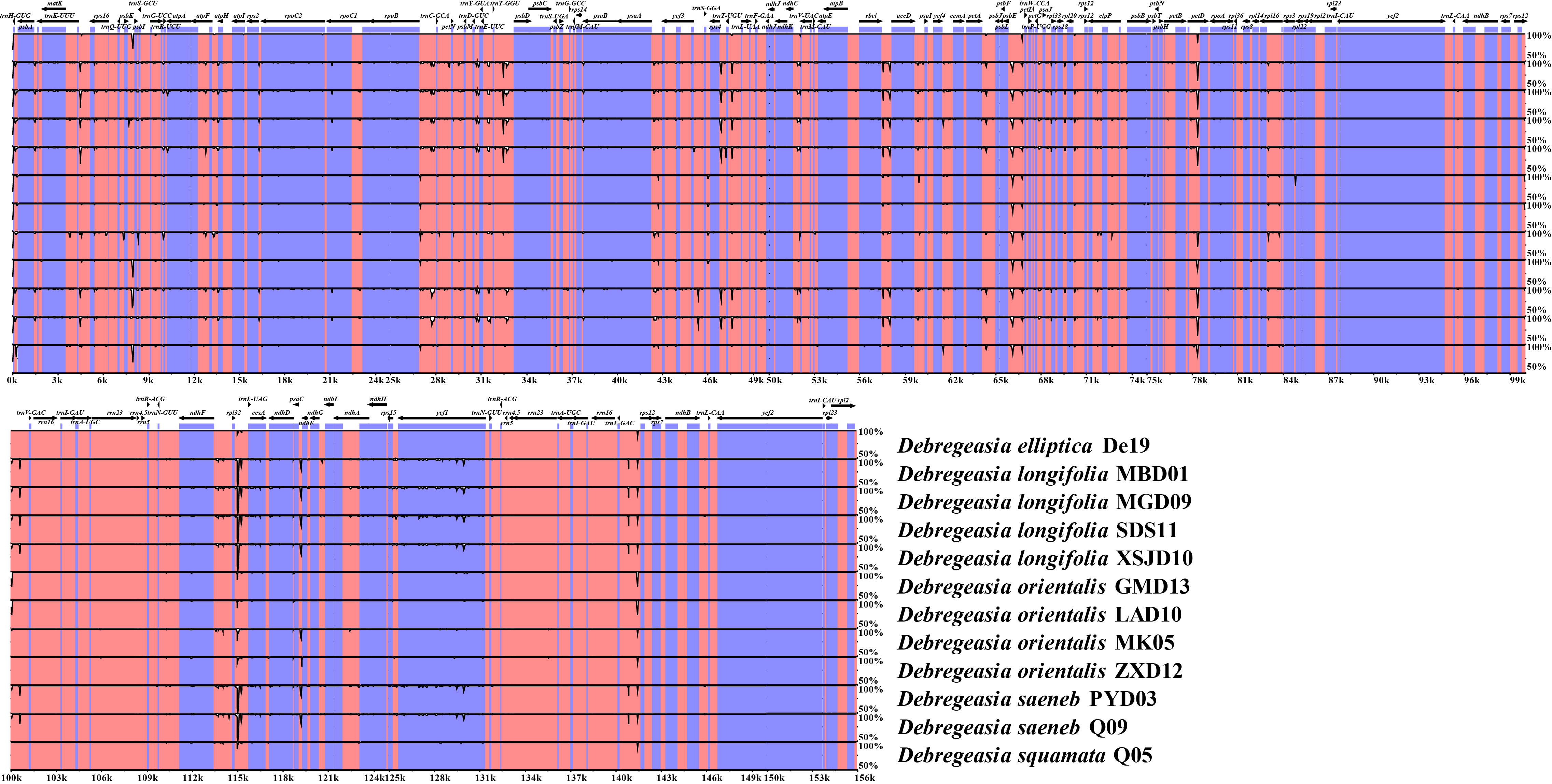

Supplement: FIGURE S1 — Comparison of the borders of LSC, SSC, and IR regions in Debregeasia, based on 13 individuals of five species. [file Data_Sheet_1.zip › supplementary files/Figure S2.tif]

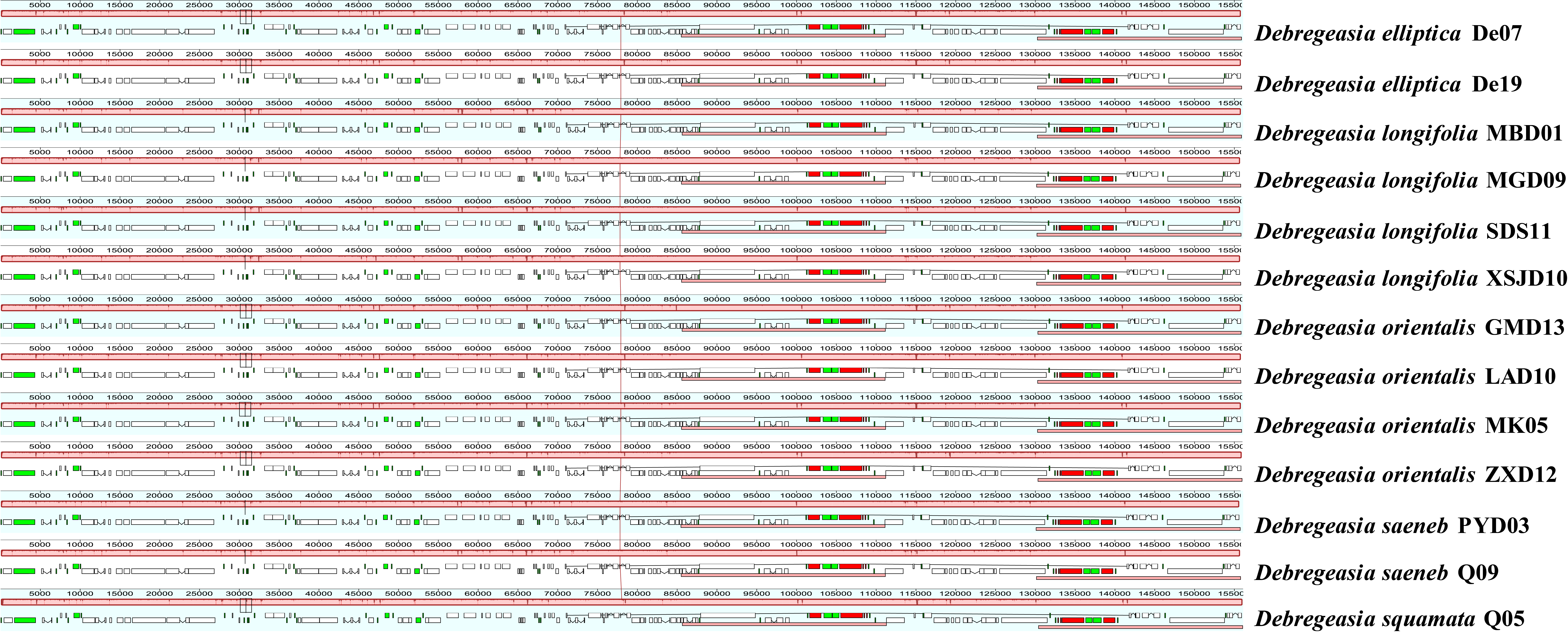

Supplement: FIGURE S1 — Comparison of the borders of LSC, SSC, and IR regions in Debregeasia, based on 13 individuals of five species. [file Data_Sheet_1.zip › supplementary files/Figure S3.tif]

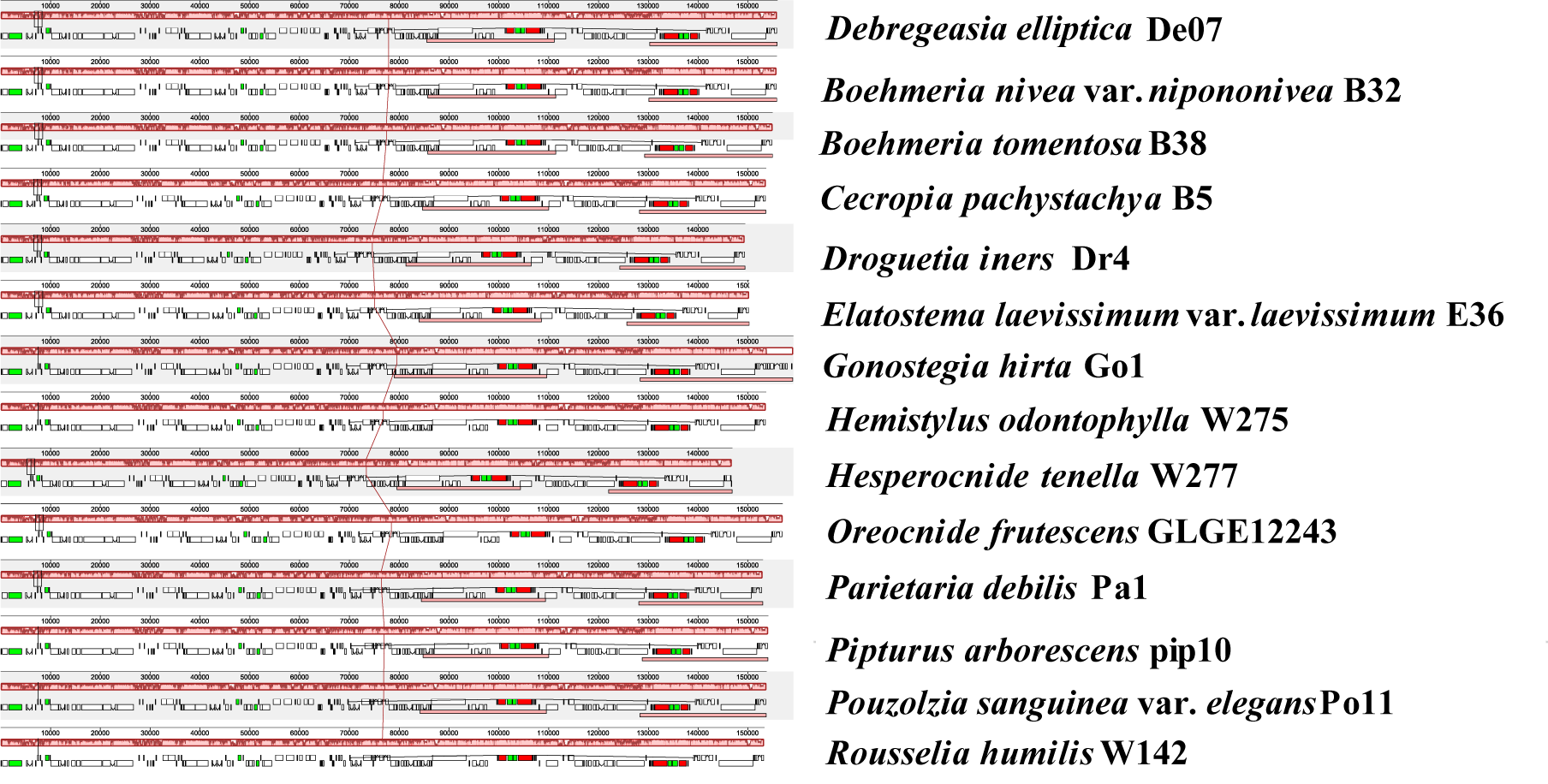

Supplement: FIGURE S1 — Comparison of the borders of LSC, SSC, and IR regions in Debregeasia, based on 13 individuals of five species. [file Data_Sheet_1.zip › supplementary files/Figure S4.tif]

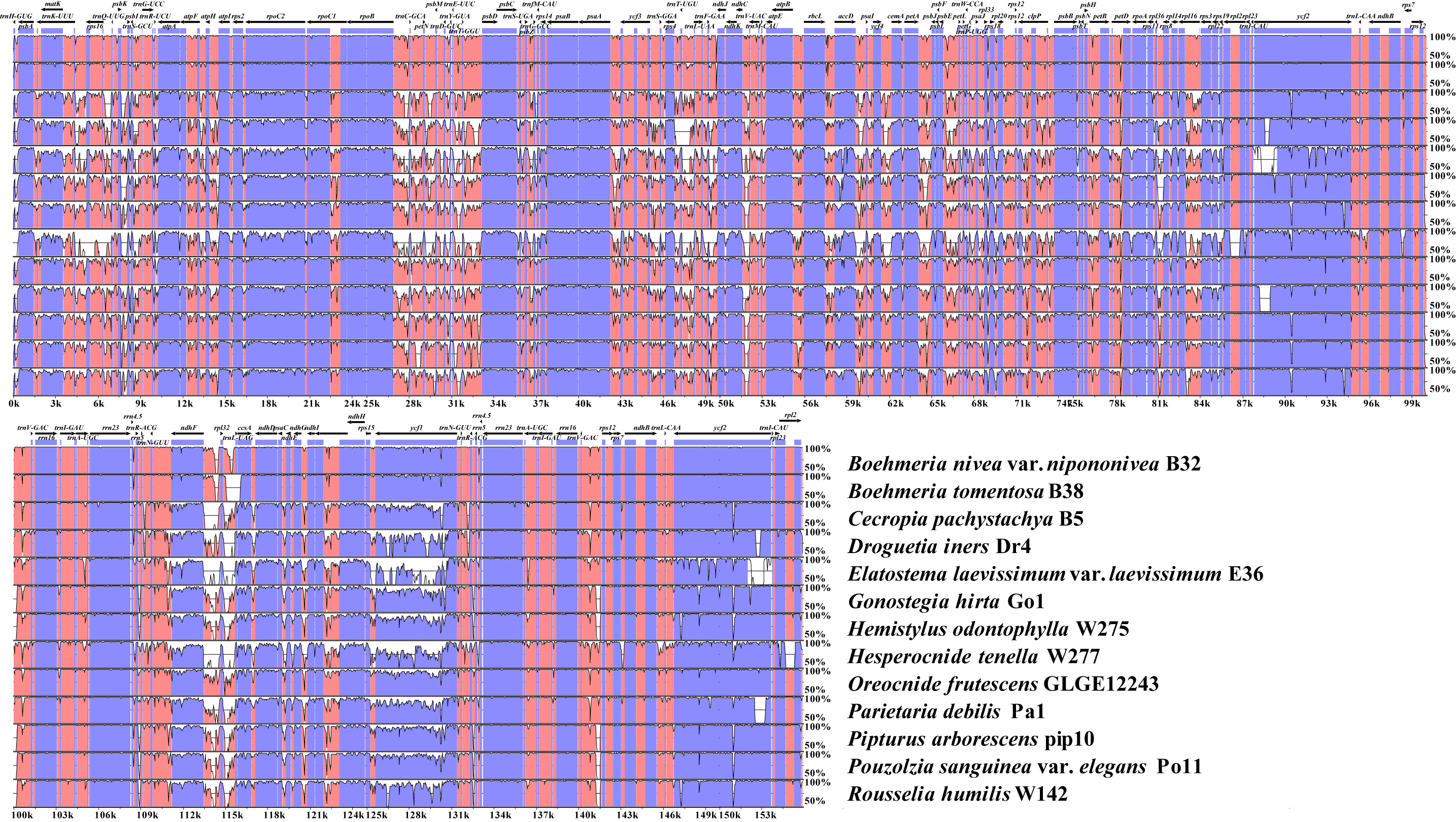

Supplement: FIGURE S1 — Comparison of the borders of LSC, SSC, and IR regions in Debregeasia, based on 13 individuals of five species. [file Data_Sheet_1.zip › supplementary files/Figure S5.tif]

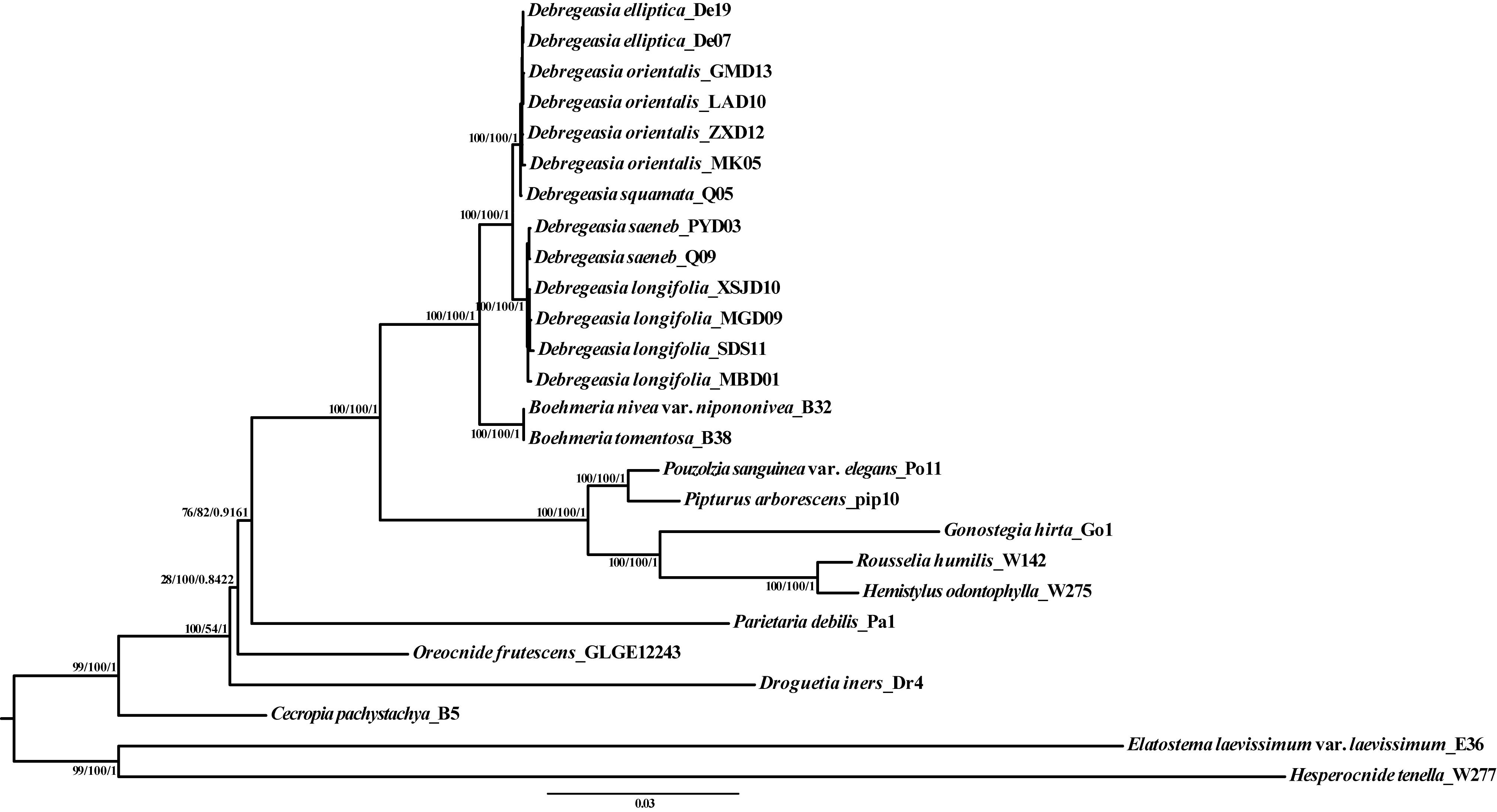

Supplement: FIGURE S1 — Comparison of the borders of LSC, SSC, and IR regions in Debregeasia, based on 13 individuals of five species. [file Data_Sheet_1.zip › supplementary files/Figure S6.tif]
